# A Review of Advance Care Planning Programs in Long-Term Care Homes: Are They Dementia Friendly?

**DOI:** 10.1155/2014/875897

**Published:** 2014-03-16

**Authors:** Abigail Wickson-Griffiths, Sharon Kaasalainen, Jenny Ploeg, Carrie McAiney

**Affiliations:** ^1^School of Nursing, McMaster University, 1280 Main Street West, Hamilton, ON, Canada L8S 4K1; ^2^Department of Health, Aging and Society, McMaster University, 1280 Main Street West, Hamilton, ON, Canada L8S 4K1; ^3^Department of Psychiatry & Behavioural Neurosciences, St. Joseph's Healthcare Hamilton, 100 West 5th Street, Hamilton, ON, Canada L8N 3K7; ^4^Geriatric Psychiatry Service, St. Joseph's Healthcare, 100 West 5th Street, Hamilton, ON, Canada L8N 3K7

## Abstract

*Background*. Persons living with dementia in the long-term care home (LTCH) setting have a number of unique needs, including those related to planning for their futures. It is therefore important to understand the advance care planning (ACP) programs that have been developed and their impact in order for LTCH settings to select a program that best suits residents' needs. *Methods*. Four electronic databases were searched from 1990 to 2013, for studies that evaluated the impact of advance care planning programs implemented in the LTCH setting. Studies were critically reviewed according to rigour, impact, and the consideration of the values of residents with dementia and their family members according to the Dementia Policy Lens Toolkit. *Results and Conclusion*. Six ACP programs were included in the review, five of which could be considered more “dementia friendly.” The programs indicated a variety of positive impacts in the planning and provision of end-of-life care for residents and their family members, most notably, increased ACP discussion and documentation. In moving forward, it will be important to evaluate the incorporation of residents with dementia's values when designing or implementing ACP interventions in the LTCH settings.

## 1. Introduction

ACP is especially relevant for persons with dementia living in LTCHs. Over time, these persons will experience progressive cognitive decline and poor health outcomes and ultimately lose their ability to communicate treatment preferences or wishes [1, 2]. Given the high prevalence of persons with dementia that reside in LTCHs in the United Kingdom and North America, discussing their wishes and treatment preferences is appropriate [1, 3]. Several programs that promote ACP in LTCHs have been described and evaluated in the literature. However, little work has been done to assess whether these programs include the consideration of values important to persons with dementia and their family members. Due to the nature of the disease and the unique needs of persons with dementia and their family members, it is important to understand which ACP programs are best suited for this population. Therefore, the purpose of this paper is twofold: first to determine the impact of the ACP programs implemented in LTCHs and second to evaluate the programs' inclusion of considering the important values of persons with dementia according to the Dementia Policy Lens Toolkit (DPLT) [4].

ACP is a process that facilitates the communication and understanding of care preferences between a person deemed to have decision-making capacity and their primary health care provider, family member(s), or substitute decision maker [5, 6]. Cantor and Pearlman [7] assert that ACP involves three components, including the consideration of health care options and expression of the person's values, communicating their wishes, and subsequent documentation. The documentation is known as creating advance directives (ADs) or a living will. Although ACP is defined as only the communication of wishes [5, 8], ADs are commonly documented as they may be used to decisively direct care in an emergency and typically carry more legal weight than discussion alone [9]. Importantly, whether ACP is documented or not in the long-term care home (LTCH) setting, discussions should be comprehensive. Residents, family members (or substitute decision maker), and health care providers may discuss the resident's thoughts on reversible conditions, the intensity of desired treatments (feeding, hydration, and medication), place of care, and naming of the power of attorney for health care [10, 11]. Overall, it is intended that ACP will result in future care that is provided in accordance with resident's preferences should they lose their decision-making capacity.

Engagement in ACP is important in LTCHs for reasons such as compliance with policy or legislation, the increasing prevalence of dying residents, and residents' desire to communicate their wishes. It is important to note that there has been greater legislative attention given to ACP [9]. In addition, some of the oldest and frailest people reside in LTCHs [12]. Consequently, about 20% of older adults die in LTCHs in the United Kingdom and Australia, and up to 29% of older Canadians die in this setting [13–15]. Also, given LTCHs residents' complex medical issues and disabilities, they may be less able to communicate their health care preferences [12, 16]. Thus, as summarized by Dobalian [17], residents may engage in ACP for many reasons including the acknowledgement of potential incapacity that may limit or eliminate their ability to express decisions and the desire for future health care treatments to be congruent with their wishes. The combination of these factors makes ACP for end-of-life care critical in this setting.

Persons with dementia are particularly suitable candidates for ACP given the nature of their disease. Dementia has been described as a terminal illness, caused by neurodegeneration, and characterized by progressive cognitive impairment [18, 19]. As the disease progresses to the terminal stage, the ability to meaningfully communicate, ambulate, or manipulate objects is severely impaired [20]. Although variation exists, the mean survival time of residents diagnosed with dementia is about seven to ten years [1]. Therefore, ACP is important, as it is expected that persons with dementia will lose their decision-making capacity and be unable to direct their care as their end-of-life approaches.

Despite the poor prognosis of persons with dementia coupled with their growing numbers in LTCHs, their health care preferences are not always known [12, 21, 22], which can lead to inappropriate palliative care and difficult decision-making for family members. Mitchell et al.'s [22] study found that persons with dementia were less likely than those with cancer to have ADs to communicate care preferences, as dementia is not always recognized as a terminal disease. Study findings have also indicated that health care providers must default to full treatment when care limiting options are unknown, and persons with dementia have received burdensome interventions, such as hospitalizations, restraint use, intravenous therapy, tube feedings, antibiotics, or life-sustaining medications [22–24]. Another consequence of no or little ACP engagement is family members having to make uninformed decisions for residents [25]. Although a lack of congruence has been found between patients' and their proxies' decisions [26], family members may be able to make more informed choices if they have engaged in ACP [27]. Therefore, given the limited survival time, eventual incapacity to make health care decisions, and noted potential for poor palliative care services and interventions, it is important to address ACP in the LTCH setting for persons with dementia.

Recognizing the importance of ACP within the LTCH setting, especially for persons with dementia, it is essential to review what programs have been used in LTCHs to promote ACP. Also, given the prevalence of persons with dementia in LTCHs, it is equally important to evaluate how these programs include the consideration of values important to persons with dementia and their families. Systematic reviews have evaluated the effectiveness of interventions designed to increase AD completion rates for adults [28] and, specifically, community-dwelling older adults [29]. Robinson et al. [30] have completed a systematic review of the effectiveness of ACP interventions for people with cognitive impairment and dementia. Also, Dening et al. [31] conducted a general review of ACP for persons with dementia to determine the key themes in the literature as well as facilitators and inhibitors for affected persons. However, an extensive literature search failed to identify a review that evaluated ACP programs in LTCHs using the DPLT to ascertain whether the values of persons with dementia were considered. Therefore, to expand on existing reviews, this paper will contribute an up-to-date review of evaluation studies that (a) focus on programs that promote ACP in the LTCH setting, (b) employ a quantitative, comparison-group study design, and (c) have an adequate description that can be critiqued using the DPLT [4]. The specific research questions addressed in this paper are the following: what are the impacts of programs used to promote ACP in LTCHs and do the programs include a consideration of the values that are important to persons with dementia and their family members?

This paper includes the following definitions for common terms. First, a* program* is defined as the processes that promote ACP through strategies, programs, or interventions, implemented in LTCHs. Second, an* impact* is defined as the reported findings from ACP program's implementation. Third,* LTCHs* are residences for older adults requiring accessible 24-hour nursing care [32]. References to nursing homes, aged-care facilities, and care homes are considered to be LTCHs. For the purposes of this paper, although a LTCH provides care for adults older than 18 years, the focus is older adults aged over 65 years. Last,* family members* are also described as substitute decision makers, where appropriate.

## 2. Methods

### 2.1. Search Strategy

In consultation with a library liaison from McMaster University's Health Sciences Library, electronic databases and key search terms for LTCHs, ACP programs, and interventions were identified. Medline, Excerpta Medica Database (EMBASE), Cumulative Index to Nursing and Allied Health Literature (CINAHL), and Ageline were searched for the literature published between 1990 and May 2013. The publications' titles and available abstracts were reviewed by one reviewer (Abigail Wickson-Griffiths) for relevant literature. Also, websites for the Physicians Orders for Life-Sustaining Treatment Paradigm (POLST), Gold Standards Framework in Care Homes, and Promoting Excellence in Palliative Care in Nursing Homes were accessed for further details of these programs. Subsequently, project or evaluation lists contained within the websites were reviewed to identify any further publications not yielded in the initial search.

### 2.2. Study Inclusion Criteria

The following four inclusion criteria were prerequisites for study review. First, studies had to evaluate a program focused on ACP and/or its components such as ADs or sharing goals of care. More broadly focused end-of-life or palliative care programs were included, given that a main outcome or objective was promoting ACP. Second, the program had to be evaluated within the LTCH setting. Third, an adequate description of the ACP program had to be available within the publication or obtainable through other means such as the authors' related work or websites. Fourth, studies had to include a quantitative design with a comparison or control group. Studies that used an uncontrolled, before-after design were excluded.

### 2.3. Critical Appraisal Tool Description and Rationale

In order to evaluate the quality of the studies, criteria from an evidence-based nursing textbook that detailed methods to evaluate health care interventions were used [38]. Overall, the method was selected because of the high regard for the contributions made to evidence-based nursing by the editors [39]. In addition, a number of evaluation guides were offered in the textbook; however, the criteria for health care interventions were most congruent with ACP programs in LTCHs. Descriptions and rationales were provided for each evaluation criterion that facilitated and guided the critique of the studies.

### 2.4. DPLT Description and Rationale

There are 11 criteria in MacCourt's [4] DPLT that are designed to collectively aid in the evaluation of policies, guidelines, and/or programs that affect people with dementia and their family members. However, for the purpose of this paper, only the sixth criterion was used to evaluate ACP programs: “Does the policy, guideline, program consider values important to those affected by dementia?” (p. 4). According to MacCourt [4], the sixth criterion considers that “respect for the person with dementia, their needs, their values, and their choices” (p. 8) is important. MacCourt [4] suggests that an evaluator should score each category with a yes or no rating and review the overall results to determine the suitability of the program. This tool was specifically selected because it was comprehensively developed from the efforts of multiple stakeholders including researchers, clinicians, and persons with dementia and their caregivers [4]. In addition, it addresses key aspects of dementia-friendly care, such as accessibility, and person-centred and relationship-based care [4].

## 3. Results

### 3.1. Results of Literature Search

The search returned 6145 sources. Upon completion of the title and abstract review, a total of 16 ACP programs were identified in a total of 26 articles. As indicated by DiCenso and Guyatt [38], randomized control trials (RCTs) are appropriate to evaluate intervention studies. However, only two RCTs were identified [33, 35]. Given the dearth of highly rigorous studies in this body of literature, studies were also included that indicated the use of quantitative design with at least one comparison or control group. Subsequently, four additional studies met this inclusion criterion [5, 34, 36, 37]. For the remaining, 18 studies did not meet the criteria [40–57], one involved a cointervention [58], and one did not provide adequate detail of the ACP component of the program [59]. Therefore, six ACP programs were evaluated to identify their impacts.

The methods and context of these reviewed studies are summarized in Tables 1 and 2.

### 3.2. Impacts

The following presents a synthesis of the reported impact(s) for each of the reviewed programs. It is important to note that any references to statistically significant or significant impacts were determined by a reported *P* value of ≤0.05 in the respective studies. See Table 3 for a summary of the reported impacts.

#### 3.2.1. Let Me Decide

Let Me Decide is a specific ACP program that enables residents or their substitute decision makers to, first, understand their treatment options in life-threatening, reversible, and irreversible health conditions and, second, record their wishes in ADs for resuscitation, feeding, and level of care [10, 60]. Molloy et al. [35] conducted a randomized control trial (RCT) to evaluate the systematic implementation of the Let Me Decide program in three LTCHs. The study reported significantly fewer hospitalizations as well as a less average cost per resident in the program LTCH sites when compared to the controls [35]. At the completion of the study, residents in LTCH sites implementing the program had completed more ADs (71%) than the controls (57%). Of the residents who completed ADs, 89% were the Let Me Decide directive in the LTCH sites employing the program and 71% were the do-not-resuscitate directive in the control sites [35]. It was inferred that the Let Me Decide directive would be more comprehensive and, thus, better able to communicate specific resident wishes than a do-not-resuscitate directive. Overall, increased ACP documentation, fewer hospitalizations, and less resource were the main impacts.

#### 3.2.2. Let Me Talk

Let Me Talk is a four-step ACP program that seeks to explore residents' prior life experiences and values before expressing their health care preferences [5]. The focus of the program shifted from the more traditional approach of developing ADs to the exploration and sharing of residents' values in the planning process. Four sequential individual interviews with residents were conducted exploring their life stories, illness narratives, life views, and, finally, end-of-life care preferences [5]. Chan and Pang's [5] multisite, quasi-experimental study showed that, over time, residents in the Let Me Talk program had significantly more stable health care preferences than those in the comparison group [5]. Better quality of life scores were found for residents that engaged in the program [5]. The participants in the Let Me Talk group were significantly more likely to share their health care preferences with family or caregivers than those in the comparison group [5]. Overall, the impact of this program was increased knowledge of residents' ACP for staff and families, improved quality of resident life, and stability of health care choices.

#### 3.2.3. Social Work Strategy to Enhance ACP Documentation

The program that was used to enhance ACP discussion and documentation in Morrison et al.'s [34] study was based on the educational material provided in module one of the Education for Physicians on End-of-Life Care (EPEC) course [61], (S. Morrison, personal communication, May 11, 2011). A controlled clinical trial was used to evaluate resident outcomes after two social workers received the EPEC training and engaged in structured methods of promoting and documenting ACP. Residents and/or their substitute decision makers were invited to share their health care preferences for life-sustaining treatments and place of care in the residents' current state of health and two hypothetical cases of moderate and severe dementia. Morrison et al. [34] found that residents in the program group were more likely to have specific instructions documented for resuscitation, intravenous antibiotics, artificial nutrition, and hospitalization than residents in the comparison group. In addition, the residents cared for by social workers in the program group were significantly more likely to receive care in adherence with their wishes than those in the comparison group [34]. In all, the reported impacts were increased documentation of specific ACP and adherence to residents' and family members' preferences.

#### 3.2.4. Improving Hospice Service

In an effort to improve enrolment in hospice services, Casarett et al.'s [33] RCT study evaluated the communication of residents' goals of care and suitability for palliative care to physicians. The Promoting Residents' Involvement in Decisions at End-of-Life (PRIDE) assessment tool was used to discover residents' goals of care (for comfort care and life- sustaining treatment) and suitability for palliative care (having palliative care needs) (D. Casarett, personal communication, May 11, 2011). It was reported that the residents who had their preferences and palliative care needs communicated to the physician were significantly more likely to enrol in hospice services compared to the control group, both within 30 days of the PRIDE assessment (20% versus 1%) and during the six-month follow-up period (25% versus 6%) [33]. Also, when compared to the control group, residents who participated in the program had significantly fewer acute care admissions and days in hospital [33]. Overall, impacts of this program included appropriate palliation or end-of-life care services in accordance with ACP and health care preferences.

#### 3.2.5. Palliative Care Quality Improvement Programs

Two studies employing LTCH-wide programs to improve the quality of palliative care were identified including Improving Nursing Home Care of the Dying [36] and Promoting Excellence in End-of-Life Care [37]. Both of these studies focused on reporting on the training of the LTCH staff and overall impacts of the program after implementation.

The Improving Nursing Home Care of the Dying is a multifaceted education program, which focuses on recognizing end-of-life, pain management, emotional and spiritual care, caregiver considerations, and ACP [62]. Promoting Excellence in End-of-Life also provides education on relevant palliative topics including ACP, pain, and psychosocial issues, such as bereavement and spirituality [37]. Documentation of ACP discussion increased from 4% to 17% for residents receiving the program in Hanson et al.'s study [36]. Similarly, Strumpf et al. [37] reported that residents participating in the program had significantly more advanced care plans near time of death when compared to the controls. Overall, the impact of these palliative care quality improvement strategies was increased ACP discussion documentation.

### 3.3. Evaluating ACP Programs Using the Dementia Policy Lens Toolkit

The following section provides the results of evaluating the ACP programs using the sixth criterion of the DPLT [4]. This criterion contains six main categories (with the number of subcategories indicated in the parentheses) and includes (a) respect and dignity (three), (b) self-determination and independence (four), (c) social inclusiveness/relationships/participation (five), (d) fairness and equity (two), (e) security (six), and (f) protection and risk management (seven). Each category was scored with a yes or no rating, as per the guidance provided by MacCourt [4]. In cases where a subcategory was judged to not apply to ACP programs in general, it was not included in the evaluation. The narrative section below describes where certain subcategories have been included or omitted. Please refer to the full DPLT [4] for reference.

Of note, because the Let Me Talk program evaluation did not include residents with cognitive impairment, it was not included in this component of the evaluation. The evaluation is summarized in Table 4.

Because the palliative care-quality improvement programs [36, 37] were implemented throughout the LTCHs, it was assumed that all residents, including those with dementia, would have been affected by their implementation and impacts. This assumption is based on the prevalence of persons with dementia residing in LTCHs in general.

#### 3.3.1. Respect and Dignity

The respect for and the dignity of persons with dementia were an included consideration in all of the reviewed programs. Even residents with more severe cognitive impairments could be included in the studies despite their capacity for decision-making, through their substitute decision makers. For example, when residents were assessed to lack the capacity to either make future treatment decisions and/or appoint a substitute decision maker, their next of kin (or the like) were invited to engage in the ACP programs on their behalf, in Molloy et al.'s [35], Casarett et al.'s [33], and Morrison et al.'s [34] studies. In addition, the training provided to staff in Hanson et al.'s [62] study encouraged them to include family members or substitute decision makers when engaging in ACP where residents did not have the capacity to make health care decisions. Also of note, Molloy et al. [35] indicated that residents participating in the Let Me Decide program may have been able to understand and indicate their own preferences with a Standardized Mini Mental State Exam (SMMSE) score as low as 16 out of 30. In this case, the program facilitator may have been able to help residents with cognitive impairment share their health care preferences. Therefore, these programs demonstrated respect and dignity, as they were designed to help include residents with dementia and their family members or substitute decision makers.

#### 3.3.2. Self-Determination and Independence

Most of the programs received an overall “yes” rating in this category, indicating that self-determination and independence for persons with dementia was considered. For example, residents and/or their substitute decision makers were invited to discuss care preferences on an ongoing basis in Strumpf et al.'s [37] study or if there was a change in health status in Morrison et al.'s [34] study. Molloy et al.'s [35] also included a followup with participants at the one-year mark. This helps to underscore that these residents and their substitute decision makers were encouraged to reevaluate their preferences as their health circumstances changed.

However, there were a few exceptions in this category. None of the studies explicitly included a description that promoting coping skills was facilitated; however, Strumpf et al. [37] noted that assessments for family, community, and bereavement support were included in the program. Thus, this important consideration for persons with dementia was mostly found to be lacking in description. Second, the program described in Casarett et al.'s [33] study stated that residents and family members could express their goals and preferences for comfort care and life-sustaining treatment. However, the process around actually choosing hospice care was not clear.

#### 3.3.3. Social Inclusiveness/Relationships/Participation

Overall, the five subcategories in this category were more difficult to evaluate. The subcategories that included accessing social, family, and community support networks were judged to not directly relate to the ACP programs and were, therefore, not included. Also, none of the studies explicitly described whether any barriers were removed for the residents' and/or family members' participation in the ACP program. However, it is apparent in Morrison et al.'s [34] study that attempts to identify existing but unknown substitute decision makers were made. Also, where in-person discussions with substitute decision makers were not possible, social workers engaged with this group over the telephone. Similarly, where identifiable and available, substitute decision makers were invited to participate in Molloy et al.'s [35] and Casarett et al.'s [33] studies. Additionally, the section of the PRIDE interview to determine hospice appropriateness was estimated to take between five and ten minutes to complete, making this a quick program for residents and their substitute decision makers [33].

Moreover, since all programs included persons with dementia and cognitive impairment, the subcategory of promoting a sense of mattering for persons with dementia demonstrated the inclusion of this important value. In addition, the programs employed by Hanson et al. [36] and Strumpf et al. [37] clearly indicated that spirituality was an included component in the palliative care training programs for staff. The ACP training provided to staff in Hanson et al.'s [36] study encouraged them to talk to residents and their family members about including emotional support and spirituality into their end-of-life care plans. Therefore, overall, the value of social inclusion, relationships, and participation was demonstrated through promoting a sense of mattering and supporting spirituality in the ACP programs.

#### 3.3.4. Fairness and Equity

All of the programs described were judged to be fair and equitable as they included residents with dementia and their family members. However, one limitation noted in Casarett et al.'s [33], Molloy et al.'s [35], and Morison et al.'s [34] studies was that residents who were assessed to lack decision-making capacity and did not have an identifiable substitute decision maker were excluded from participation. It is reasonable to speculate that this may also be the case in Hanson et al.'s [36] and Strumpf et al.'s [37] studies. Although logistically it would be impossible for this small group of residents to participate in the respective programs, they did not have a chance of relating their goals of care through alternative means.

#### 3.3.5. Security

It was judged that only the subcategory of being able to plan for the future was applicable in evaluating the ACP programs. Given the nature of all of the ACP programs, each program provided this option.

#### 3.3.6. Protection and Risk Management

Only the subcategory of “ensuring the preferences of people with dementia are taken into account as much as possible” [4, p.8] was included in this evaluation, and all of the programs received a “yes” rating. As noted in Morrison et al.'s [34] study, in cases where residents were unable to make health care decisions but were still deemed able to appoint a substitute decision maker, they were able to do so. Social workers also tried to determine if a substitute decision maker had been named for residents no longer able to appoint one [34]. These substitute decision makers were encouraged to share any previously expressed preferences for health care on behalf of the resident. In cases where previous discussion between the substitute decision maker and resident did not occur, they were asked to answer questions based on the residents' best interests [34]. This inclusion demonstrates considering residents preferences as much as possible, even in uncertain situations. Also of note, in Casarett et al.'s [33] study, where the resident's and substitute decision maker's responses were not congruent, both were communicated to the physician who could consider and attempt to reconcile this. Finally, as noted above, the Let Me Decide program was designed for residents with an SMMSE score as low as 16 to be able to share their health care preferences [35].

Overall, the reviewed ACP programs demonstrated important impacts including increased ACP discussion and documentation, adherence to resident and substitute decision maker wishes or preferences, reduced resource utilization (cost), increased staff and family knowledge about resident's wishes, improved resident quality of life scores, and stability of health care choices over time. With the notable exception of the Let Me Talk program [5], which was not included in the DPLT evaluation, all of the remaining programs demonstrated some inclusion of values important to persons with dementia and their family members and therefore can be considered “dementia friendly,” by that standard.

## 4. Discussion

Others have reviewed the impacts of AD completion rates [28, 29] and the effectiveness of ACP programs for persons with dementia [30] in the older adult population. However, this review contributes the evaluation of ACP programs in LTCHs, according to the sixth criterion of the DPLT [4]. Not only does this review highlight the overall “dementia friendliness” of the evaluated programs, but it also outlines the additional impacts included in the Let Me Talk program, not previously addressed in other reviews [30, 63–65].

Although decades have passed since legislation has supported AD, few highly rigorous studies have been published that evaluate ACP programs in LTCH setting. Similar to the observations in the reviews of end-of-life care, as well as palliative care interventions in LTCHs [63–65], the research dedicated to ACP programs in this setting is largely descriptive or of a weaker evaluative quality [40–57]. Also, like Robinson et al.'s [30] review, whose included studies were all in LTCHs, this review determined the impacts of the Let Me Decide [35], Improving Nursing Home Care of the Dying [36], and the intervention involving social workers to enhance ACP [34], which included ACP documentation, fewer hospitalizations, less resource use, and adherence to residents' and family members' wishes. However, in using more broad inclusion criteria, repeated and additional impacts for the LTCH setting were identified including adherence to residents' and family members' wishes [33], increased knowledge of residents' wishes for staff and families, improved quality of resident life, and stability of health care choices [5]. In addition, while other studies have reported positive impacts for LTCHs [40–57], more rigorous evaluations are needed to substantiate these findings. Also, with the exception of Molloy et al.'s [35] study, the cost or resource utilization of the ACP programs was not considered or evaluated in the reviewed studies, which could have serious implications for the implementation and use of these programs. Therefore, in moving forward, more rigorous testing through RCT or clinical control trial designs [30] and economic analysis of the reviewed programs are needed.

Also, in moving forward with evaluation of programs that focus on dementia care, or those that are implemented in LTCHs, the DPLT [4] may be considered. While no other published studies could be located that used the DPLT to guide program evaluation, the sixth criterion provided a helpful lens to evaluate the programs in terms of how they considered values important to persons with dementia. However, given the purpose of ACP program, it was difficult to use all of the categories of the sixth criterion, especially social inclusiveness/relationships/participation, security, and risk management. Additionally, being limited to the description of the reviewed ACP programs provided in the journal articles and websites, there was difficulty in fully using the sixth criterion of the DPLT [4]. A great degree of detail would be needed to use all of the subcategories, which was beyond the scope of the description provided in the evaluation studies. In the future, if LTCH personnel are trying to decide on implementing a new program, perhaps asking the authors of the potential program to complete the full DPLT would be helpful in determining if it does in fact promote excellence in dementia care.

### 4.1. Recommendations and Implications for Nursing Practice

Overall, the review indicated a variety of positive ACP impacts from mostly “dementia-friendly” programs. However, given that a main outcome of the ACP process is creating an understanding of a person's health care preferences, so that they may receive treatment that is congruent with their wishes, selecting a program that promotes this outcome, such as those evaluated by Casarett et al. [33] and Morrison et al. [34], seems most appropriate to consider. However, due to the varying characteristics, capacity, and legislative requirements, it is recognized that no one ACP program will meet the needs of every LTCH, its residents, and/or family members. Careful consideration of the most appropriate program for each LTCH or corporation is warranted. The following provide some additional points of consideration.

- It is important that the program provide direction around engaging in a comprehensive ACP discussion and perhaps subsequent documentation [10]. The Let Me Decide [35], social workers [34], and Improving Nursing Home Care of the Dying [36] programs provide tools (available to authors) for guiding discussion and providing information around a variety of health care problems. The comprehensiveness of the discussion is important in creating an understanding of a person's preferences, and therefore selecting a program that offers specific guidance around discussion topics is recommended.
- The palliative and end-of-life care quality improvement programs are recommended for consideration because of their multifaceted design. These quality improvement programs have demonstrated that they are not only a useful tool in improving ACP discussions and documentation but also provide the education, training, and guidance to staff about how to provide high-quality palliative, end-of-life care [36, 37]. Given that a palliative approach to end-of-life care for persons with dementia has been recognized as a standard of care [1], these programs will more comprehensively and appropriately address both ACP and the provision of end-of-life care for residents, especially for those with dementia. The sustainability of the ACP program is also important to consider. In both Casarett et al.'s [33] and Chan and Pang's [5] work, research personnel were used to engage in the ACP program with residents and families. Therefore, it is unknown whether staff members of the LTCH would be able to continue employing these programs and produce the same impacts, which is considered a limitation of these evaluations. Also, given the staff turnover rate in LTCHs [41], a lack of attention to providing end-of-life care may result, if persons in leadership positions do not value the programs.
- Before selecting any ACP program, its evaluation using all of the applicable criteria from the DPLT [4] is recommended. This will help to ensure that the program considers all facets of dementia care, beyond the consideration of values important to persons with dementia and their families. In recognizing that persons with dementia should be included as much as possible in decision-making, it is essential to consider selecting a program that will allow most residents with cognitive impairment to participate. Should decision-making capacity be found to be lacking, selecting programs that explicitly involve family members and substitute decision makers involvement is encouraged [33–37].

### 4.2. Review Limitations

A few limitations of this paper are noted. First, the literature search program could have been expanded to include the reference lists of the reviewed studies. Second, due to the over 5000 sources yielded from the search strategy that were reviewed by only one person, there is a possibility that relevant articles may have been missed in the title and abstract review. Finally, the descriptions of the ACP programs provided in the reviewed studies or related websites may not have provided comprehensive details to fully appreciate every facet of their content and implementation. Therefore, the application of the DPLT was limited to the available description.

## 5. Conclusion

In conclusion, the implementation of ACP programs that include the consideration of values important to persons with dementia and their families is timely. While six unique programs have been identified, five of which can be considered “dementia friendly,” LTCHs should select the program that will best meet their identified needs and desired impact.

## Figures and Tables

**Table 1:** Characteristics of the evaluated studies.

Study characteristic	Casarett et al. [33]	Chan and Pang [5]	Morrison et al. [34]
Study design	Randomized controlled trial	Quasi-experimental	Controlled: before and after study
Setting	United States	Hong Kong	United States
	Three LTCHs	Four LTCHs	One LTCH
Sample size			
Program participants	(*n* = 107)	(*n* = 42)	(*n* = 43)
Control participants	(*n* = 98)	(*n* = 36)	(*n* = 96)
Cognitive impairment mentioned	Yes	Yes (resident with cognitive impairment not included)	Yes
Program description	Identify care preferences using PRIDE assessment and communicate to physician for referral to palliative/hospice care	Let Me Talk (interviews with residents exploring values and care preferences)	ACP training for two social workers using Education for Physicians on End-of-Life Care; structured discussion and documentation of ACP
Control/comparison	Did not communicate PRIDE assessment to physician	Care as usual	Care as usual from social workers; research associate talked to participants about health care preferences but did not record them in the medical record
Research staff involvement in intervention or comparison	Apparent	Apparent	Apparent

PRIDE: Promoting Residents' Involvement in Decisions at End-of-Life; LTCH: long-term care home; LTCHs: long-term care homes; ACP: advance care planning.

**Table 2:** Characteristics of the evaluated studies.

Study characteristic	Molloy et al. [35]	Hanson et al. [36]	Strumpf et al. [37]
Study design	Randomized controlled trial	Controlled: before and after study	Prospective study with control group
Setting	Canada	United States	United States
	Six LTCHs (three interventions)	Nine LTCHs (seven interventions)	Six LTCHs
Sample			
Strategy participants	(*n* = 527)	(*n* = 345)	(*n* = 4 LTCHs)
Control participants	(*n* = 606)	(*n* = 112)	(*n* = 2 LTCHs)
Cognitive impairment mentioned	Yes	Yes	Yes
Program description	Training provided to health care facilitators to implement Let Me Decide (AD) program in three LTCHs	The Improving Nursing Home Care of the Dying (develop palliative care teams, education for staff around palliative care)	Promoting Excellence in End-of-Life (develop palliative care teams in two LTCHs; educational training and support provided to LTCH staff and program implemented)
Control/comparison	Care as usual	Care as usual	Care as usual; new pain and advanced care policies introduced from corporation during the study period
Research staff involvement in intervention or comparison groups	Not apparent	Not apparent	Not apparent

ACP: advance care planning; LTCH: long-term care home; LTCHs: long-term care homes; ADs: advance directives.

**Table 3:** Summary of the reported impacts of the ACP strategies.

Impacts	Casarett et al. [33]	Chan and Pang [5]	Hanson et al. [34]	Molloy et al. [35]	Morrison et al. [34]	Strumpf et al. [37]
Increased ACP activities discussion	X	X	X	X	X	X*
Increased ACP discussion documentation			X*	X		
Specific ACP documentation (e.g., hydration, antibiotics, etc.)				X	X*	
Adherence to resident/substitute decision maker wishes	X*				X*	
Greater knowledge of ACP among residents						
Greater knowledge of ACP among family members		X*				
Greater knowledge of resident's ACP among LTCH staff		X*				
Reduced hospitalization	X*			X*		
Increased satisfaction with care	X*					
Stability of treatment preferences		X*				
Improved quality of resident life		X*				
Reduced resource use				X*		

X: impact noted; X*: statistically significant impact (*P* ≤ 0.05); LTCH: long-term care home; ACP: advance care planning.

**Table 4:** Evaluation of advance care planning programs using the sixth criterion of the Dementia Policy Lens Toolkit.

Criteria	Casarett et al. [33]	Hanson et al. [36]*	Molloy et al. [35]	Morrison et al. [34]	Strumpf et al. [37]*
Respect and dignity					
Is the policy/program flexible enough to respond to the uniqueness of each individual?	Y	Y	Y	Y	Y
Are people with dementia and their families portrayed positively?	Y	Y	Y	Y	Y

Self-determination and independence					
Does the policy/program:					
Provide opportunities to make choices?	Y	Y	Y	Y	Y
Reflect knowledge of what is important to the person?	Y	Y	Y	Y	Y
Promote coping skills/strengths?	U	U	U	U	U
Promote and support option and informed choices for people with dementia and their caregivers at each phase of the disease/transition point?	N	U	U	Y	Y

Social inclusiveness/relationships/participation					
Are any barriers to the participation of people with dementia and their families removed?	Y	U	Y	Y	U
Is spirituality supported?	N	Y	U	U	Y
Is a sense of mattering facilitated?	Y	Y	Y	Y	Y

Fairness and equity					
Are the procedures and criteria inherent in the policy/guideline/program fair and reasonable?	Y	Y	Y	Y	Y
Does it consider individual versus collective needs?	Y	Y	Y	Y	Y

Security					
Does the policy/program:					
Provide the security of being able to plan for the future (appropriate house and services, death)?	Y	Y	Y	Y	Y

Protection and risk management					
Does the policy/program:					
Ensure that the preferences of people with dementia are taken into account as much as possible?	Y	Y	Y	Y	Y

U: unclear; Y: yes; N: no; *LTC home-wide strategy assumed that all residents are eligible for participation.

Criteria from MacCourt [4].
